# The Thermal Activation of Serpentinite from the Zhitikarinsky Deposit (Kazakhstan)

**DOI:** 10.3390/molecules29184455

**Published:** 2024-09-19

**Authors:** Abdrazak Auyeshov, Kazhymuhan Arynov, Asem Satimbekova, Chaizada Yeskibayeva, Kurman Alzhanov

**Affiliations:** 1 Scientific Research Laboratory “Applied Chemistry”, M. Auezov South Kazakhstan University, Shymkent 160012, Kazakhstan; 2 “Institute of Innovative Research and Technology” LLP, Almaty 050010, Kazakhstan

**Keywords:** X-ray phase analysis, thermal activation, serpentinite, brucite, periclase

## Abstract

Changes in the phase composition of serpentinite from the Zhitikarinsky deposit (Kazakhstan) were studied during heat treatment in the temperature range of 105–800 °C in order to determine the phase that leads to an increase in the reactivity of serpentinite during interactions with acids. In accordance with this, the task was set to consider the influence of temperatures (105 °C, 600 °C, 660 °C, 725 °C, 750 °C and 800 °C), selected according to the extreme points of the main transformations from its derivatogram, on changes in the phase composition. The effects of heat treatment on the phase composition and reactivity of serpentinite during interactions with sulfuric acid were studied and discussed using the diffraction patterns of serpentinite heat treated at selected temperatures for 1 h and using methods of thermodynamic evaluation of the possible reactions in the temperature range of 350–750 °C, as well as establishing the nature of their interaction with sulfuric acid under identical conditions. It has been shown that, in the range of 600–750 °C, the crystal lattice of the structural composition of serpentinite is completely destroyed with the formation of not only magnesium silicates but possibly also magnesium oxide, which leads to the thermal activation of serpentinite in relation to interactions with acids and the improvement of the technological process of extracting magnesium from it. The greatest increase in reactivity was found in serpentinite samples heat treated at temperatures of 725 °C and 750 °C, which can be recommended for the thermal activation of serpentinite before using acid methods to improve the technological performance of the process of extracting magnesium from serpentinite.

## 1. Introduction

Serpentinites have been the subject of numerous studies due to their physico-chemical properties and the presence of relatively high concentrations of magnesium and silicon. Relatively, a large amount of research and development is devoted to finding a technology for extracting useful components from serpentinite in the form of industrially important magnesium-containing elements [1,2], which have numerous applications in various industries.

A large deposit of chrysotile ore is also located on the territory of the Republic of Kazakhstan. The Zhitikarinsky field is located on the eastern slope of the Southern Urals, which structurally represent part of the Ural Shield, which is the eastern edge of the East European Plate. The chemical composition of chrysotile ore is mainly serpentinite Mg_3_Si_2_O_5_(OH)_4_, brucite Mg(OH)_2_ and magnetite Fe_3_O_4_. Serpentinites have been isolated within the main deposit; they include lizardite, antigorite and chrysotile. Serpentinites with small cores of peridotites or dunites have also been distinguished, up to 60% of the rock volume is serpentinites [3].

Currently, the Zhitikarinsky deposit is operated by Kostanay Minerals JSC. The capacity of the enrichment complex for the production of chrysotile is 250 thousand tons/year. The chrysotile industry is going through difficult times—this is due to both an anti-asbestos movement in the world, which has been going on for more than 30 years, and a decrease in chrysotile consumption, leading to a decrease in the sales market and the emergence of alternative materials replacing chrysotile cement products. The market economy and fierce competition in the industry for sales markets, for many mining companies in the world, including in Kazakhstan, determine and put on the agenda topical issues such as the conservation and rational use of resources. The rational use of chrysotile raw materials includes not only the production of useful substances but also environmental content. One of the alternative ways to keep up with the times is the diversification of production, improving its competitiveness. The chrysotile industry in Kazakhstan is also subject to change, as the diversification of the main goods of production creates new jobs and new products.

It is obvious that, for the introduction of innovation, it is important to develop new methods for processing Kazakhstani serpentinite, as well as serpentinite-containing waste from the production of chrysotile, of which about 250 million tons have been accumulated over 65 years of operation of the deposit. The chrysotile fiber residues contained in these wastes can pollute the air, causing serious harm to human health and the environment [4]. It should be noted that Kazakhstani serpentinites and waste from the operating company for the extraction and enrichment of chrysotile remained poorly studied for a long time with regard to their processing and the possibility of using them in obtaining industrially important magnesium and silicon compounds necessary for the development of the national economy of Kazakhstan. Since there are no high-quality dolomite deposits in Kazakhstan, exploratory research aimed at developing practical technologies for processing serpentinite and serpentinite waste in order to use them as a source of magnesium is becoming relevant for the further economic development of the country.

A study initiated to establish the initial characteristics of the serpentinite and mining waste showed that they have almost the same chemical composition [5,6,7]. This implies that both natural serpentinite and serpentinite waste from the processing of chrysotile ore can be used to achieve this goal. Since the rational use of chrysotile raw materials includes not only the production of useful material but also environmental content, it was proposed in the research to pay more attention to the problem of recycling chrysotile production waste.

Various reagents are used to extract magnesium from serpentinite [8,9,10]. An analysis of the scientific literature in this line has shown that, along with the advantages of each reagent or method, there are also disadvantages that limit their application in practice. The results of our comprehensive study of the problems of serpentinite processing, covering the entire hydrometallurgical process, including aspects of dissolution [11], purification and extraction of magnesium [12], and the ecological aspects [13], indicate the need for further research to explore alternatives to the effective use of serpentinite waste of varying nature as a source for obtaining magnesium-containing products. In this regard, one of the promising alternative ways to improve the technological conditions and increase the leaching rate, in our perspective, is the thermal activation of serpentinite before acid leaching, proposed in works [14,15]; by increasing its reactivity compared to the original (uncalcified serpentinite), high degrees of magnesium extraction from serpentinite have been achieved.

It has also been noted that the mechanism of mineral destruction during thermolysis does not depend on the structural varieties of serpentine (chrysotile, lizardite or antigorite), which is primarily due to the ratio and distribution of ortha- and metasilicate anions in the silicate layer [16]. However, in order to fully substantiate the possibility of using serpentinite as a source of magnesium and its compounds, it will be necessary to subject samples of various deposits, including the Kazakh Zhitikarinsky deposit, to similar studies. Unlike others, in this work, the main focus was on establishing the reasons leading to an increase in the reactivity (activation) of serpentinite heat treated at certain temperatures in relation to its interactions with acids.

It is expected that the continuation of this kind of research will expand the accumulated knowledge and data in the field of studies of the thermal behavior and acid decomposition of serpentinites from various deposits. They will be useful in the search for effective industrial applications of serpentinite and waste from the processing of chrysotile ore.

The aim of this work was to determine how the temperature of the heat treatment of serpentinite (Zhitikarinsky deposit) in the temperature range of 105–800 °C, selected according to the extreme regions (points) of its derivatogram, affects the state of its phase composition in relation to its reactivity during interactions with acid, as well as the characteristic indicators of the process of its dissolution in sulfuric acid.

The results of the X-ray phase analysis of serpentinite heat treated at various temperatures (105 °C, 600 °C, 660 °C, 725 °C, 750 °C and 800 °C), the visual observation of the processes of its dissolution in a sulfuric acid solution and the thermodynamic assessment of the probability of reactions occurring in serpentinite during thermal and acid treatment were studied and discussed.

## 2. Results and Discussions

The chemical (elemental) composition of the used serpentinite, in % mass was as follows: Mg—25.9; Si—17.64; Fe—3.44; Al—0.47; Ca—0.42; Cr—0.24; Mn—0.17.

The conditions for the thermal activation of serpentinite and the indicators are provided in Table 1.

Calcination temperatures in the I and II regions: at 105 °C, adsorbed moisture was removed (1.15%); in the temperature range 350–450 °C, brucite decomposed, which is always present in a small amount in the composition of serpentinite (2.58%) with the formation of magnesium oxide and water. In the III region, 600 °C to 725 °C, dehydroxylation occurred with the gradual loss of the chemically bound water of serpentinite, which is expressed by the general formula Mg_3_Si_2_O_5_(OH)_4_. In the IV region, in the range of 750–800 °C, interphase processes occurred with the formation of forsterite.

**Figure 1:**
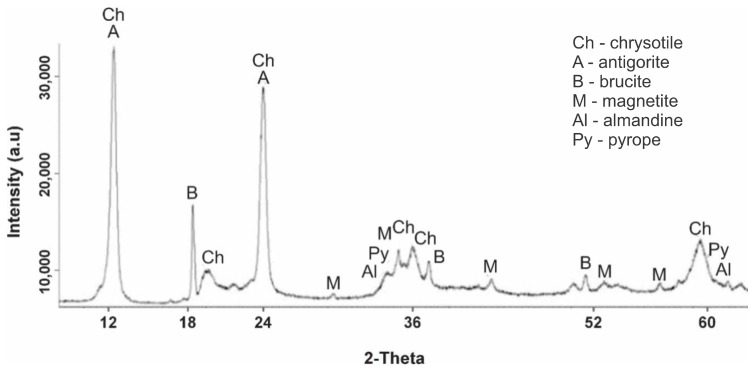
Diffractogram of the initial dry serpentine (sample No. 1, t = 105 °C, mass loss of 1.15%).

Figure 1 shows a diffractogram of serpentine sample No. 1, heat treated at 105 °C. The diffraction pattern clearly shows the peaks of the interplanar spacing (IPS) of chrysotile Mg_6_[Si_4_O_10_](OH)_8_ with values of d/n = 7.38; 4.619; 3.661; 2.487; 2.141; 1.53 Å and a small number of overlapping IPS peaks of antigorite with d/n = 7.30; 3.63; 2.52 Å, which are characteristic of the serpentine phase. The diffractogram also shows overlapping peaks of the interplane distance of a small amount of antigorite d/n = 7.30; 3.63; 2.52 Å and a small amount of magnetite Fe[Fe_2_O_3_] d/n = 2.99; 2.541; 2.098; 1.710; 1.612 Å. Very weak peaks of the interplane distance of pyrope—Mg_3_Al_2_[SiO_4_]_3_—were found at values of d/n = 2.92; 2.69; 1.50 Å and peaks of almandine—Fe_3_Al_2_[SiO_4_]_3_—occurred at d/n = 2.90; 2.60; 1.51 Å. Thus, according to the X-ray phase analysis, the following observations were made: the main phase was serpentinite (chrysotile and antigorite) and brucite was also present; a small amount of magnetite and an insignificant amount of pyrope and almandine were also observed.

**Figure 2:**
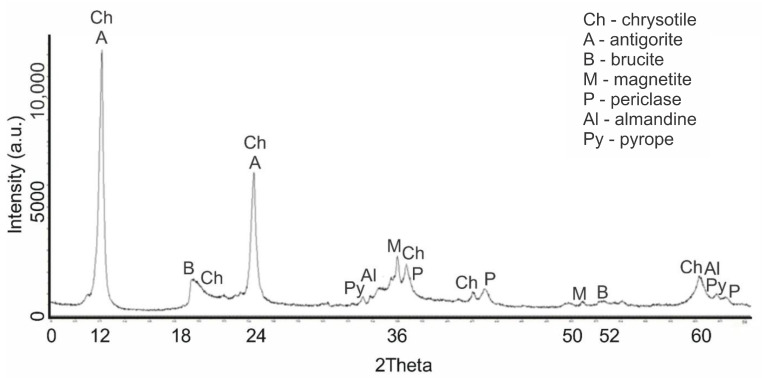
Diffractogram of serpentinite (sample No. 3, 600 °C, mass loss of 5.24%).

The diffraction patterns of samples No. 2 (400 °C) and No. 3 (600 °C) have practically identical patterns; therefore, the diffraction pattern of sample No. 3 (600 °C) was studied in more detail. The calcination of serpentinite at a temperature of 600 °C (sample No. 3) led to the decomposition of one of the main phases—brucite—which was detected on the diffractogram of the initial serpentinite at IPD values d/n = 4.77; 2.365; 1.794 Å (Figure 2). At the same time, the distance between the peaks of the interplane phase—periclase (MgO), which is a product of the decomposition of brucite—increased at values of d/n = 2.43; 2.108; 1.48 Å. The diffraction characteristics of serpentinite (chrysotile, antigorite) and magnetite did not change; their characteristic peaks of the interplanar distance were recorded without any change. In this sample, the peaks of the interplane distance of the pyrope (Mg_3_Al_2_[SiO_4_]_3_) d/n were more clearly outlined at 2.92; 2.69; 1.50 Å and those of almandine (Fe_3_Al_2_[SiO_4_]_3_) at d/n = 2.92; 2.69; 1.50 Å.

**Figure 3:**
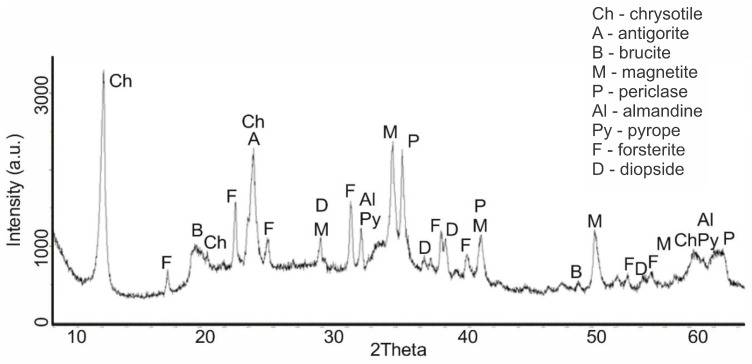
Diffractogram of serpentinite (sample No. 4, 660 °C, mass loss 11.38%).

An increase in the calcination temperature of serpentinite (sample No. 4, 660 °C) was accompanied by significant phase changes. At the same time, serpentinite underwent a significant change, which can be seen on the diffractogram by reducing the intensity of IPD peaks at values d/n = 7.38; 3.661; 2.487; 1.53 Å. Two new phases appeared—forsterite Mg_2_[SiO_4_], with IPD values of d/n = 3.875; 3.47; 2.753; 2.497; 2.441 Å and diopside CaO∙MgO∙2SiO_4_ with d/n = 2.99; 2.894; 2.56; 2.04 Å (Figure 3). At this temperature, the peaks of serpentinite did not disappear on the diffractogram; they were completely recorded, although their intensity decreased noticeably.

**Figure 4:**
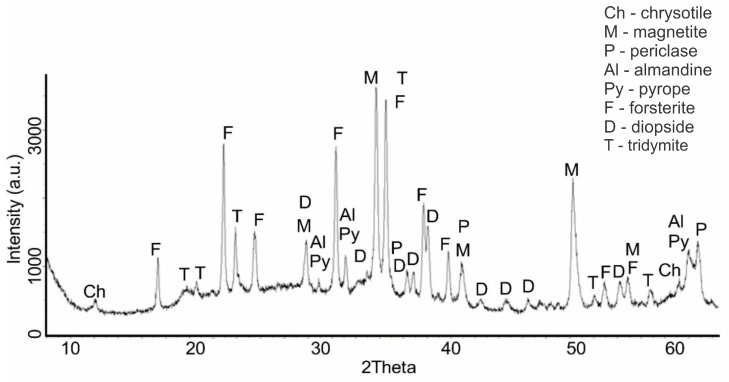
Diffractogram of serpentinite (sample No. 5) calcined at 725 °C (mass loss of 13.34%).

An increase in the calcination temperature of serpentinite to 725 °C (sample No. 5) was accompanied by the completion of the destruction process of the main phase of the serpentinite mineral. On a diffractogram of the intense characteristic IPD of serpentine d/n = 7.38(10); 3.661(10); 2.487(10); 1.53(10) Å, only one was recorded, with a small tooth at the value d/n = 7.38 Å (Figure 4). Of the new ones, a phase—tridymite (SiO_2_)—appeared, with IPD values of d/n = 4.39; 4.12; 3.73 Å, as well as all the previous phases that appeared at 660 °C with the following characteristic values of IPD: a clearly prescribed forsterite d/n = 3.875; 3.47; 2.753; 2.497; 2.441 Å, magnetite d/n = 2.99; 2.541; 2.097 Å, diopside d/n = 2.99; 2.894; 2.56; 2.04 Å, periclase d/n = 2.431; 2.108; 1.48 Å, pyrope d/n = 2.92; 2.69; 1.50 Å and almandine (Fe_3_Al_2_[SiO_4_]_3_) d/n = 2.92; 2.69; 1.50 Å. Thus, in the composition of serpentinite calcined at 725 °C, forsterite and magnetite became the main phases. The background of the diffractogram had many teeth, but they were more or less smoothed.

**Figure 5:**
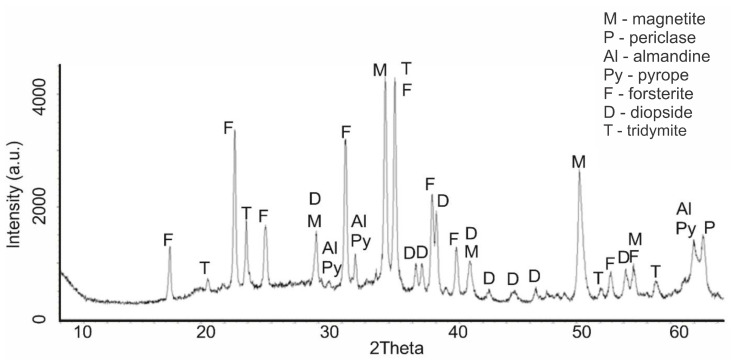
Diffractogram of serpentinite (sample No. 6) calcined at 750 °C (mass loss of 13.77%).

The calcination of serpentinite at a temperature of 750 °C (sample No. 6) was accompanied by the completion of the change in its phase composition, which began at 660 °C (Figure 5). The diffraction characteristic peaks of serpentinite completely disappeared in the diffractograms, and the characteristic peaks of phases that had already formed at the temperature of 725 °C remained, with values of the interplane distance as follows: forsterite d/n = 3.875; 3.47; 2.753; 2.497; 2.441 Å, magnetite d/n = 2.99; 2.541; 1.710 Å, diopside d/n = 2.99; 2.894; 2.56; 2.04 Å, periclase d/n = 2.431; 2.108; 1.48 Å, tridymite d/n = 4.39; 4.12; 3.7 3 Å, pyrope d/n = 2.92; 2.69; 1.50 Å and almandine d/n = 2.92; 2.69; 1.50 Å. This diffractogram was characterized by the absence of the serpentinite phase.

**Figure 6:**
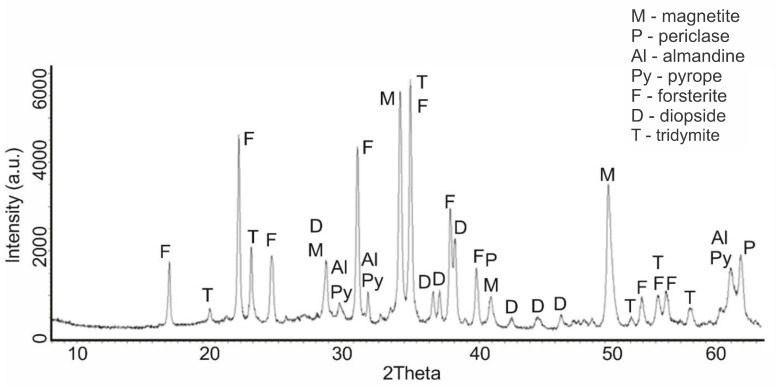
Diffractogram of serpentinite (sample No. 7) calcined at 800 °C (mass loss of 13.92%).

An increase in the calcination temperature of serpentinite to 800 °C (sample No. 7) did not introduce phase changes in the composition of the calcined sample (Figure 6). The phase composition of sample No. 7 contained the same phases—forsterite, magnetite, diopside, periclase, tridymite, pyrope and almandine, which had already formed at the temperature of 750 °C. Thus, the calcination temperature range of 750–800 °C, in which the mass loss was 13.34–13.92%, which is close to the theoretical water content (13.04%), was the completion of the phase and structural transformations of serpentinite.

In recent studies [17], it is often noted that thermal activation of serpentine before acid treatment has a number of advantages in carrying out the processes of leaching serpentinite with acids in order to extract magnesium from it. As an added effect of preliminary heat treatment during the leaching of serpentine by acidic methods, an increase in their reactivity compared to non-thermally treated ones is a given, due to the violation of the compact and stable structure of serpentine during calcination [18].

The study of the diffractogram of the initial serpentinite (Figure 1, sample No. 1) heat treated at 105 °C shows that the natural chrysotile ore of this deposit, in addition to the main serpentinite phase (antigorite, lizardite, chrysotile), contains free brucite Mg(OH)_2_, magnetite Fe(Fe_2_O_3_), small amounts of pyrope Mg_3_Al_2_[SiO_4_]_3_ and almandine Fe_3_Al_2_[SiO_4_]_3_.

Of these discovered minerals, when fired to 600 °C, only brucite underwent dehydration transformation, which is associated with the appearance of characteristic periclase reflexes. There were no other changes in the diffractogram. At the same time, the type of diffractogram (Figure 1, sample No. 2), if we do not take into account the weak reflexes of magnetite, pyrope and almandine, completely copied the diffractograms of synthetic Mg_3_Si_2_O_5_(OH)_4_, given in [19]. This discovered fact indicates that, when fired to a temperature of 600 °C, the processes associated with the dehydroxylation of Mg_3_Si_2_O_5_(OH)_4_ and the destruction of its structure (the crystal lattice of the silicate layer) apparently do not occur yet. A mention of maintaining the integrity of magnesium silicate links during the firing of serpentinite up to 600 °C is given in [16]. A significant change in the phase composition occurred when firing serpentinite at 660 °C; there was a noticeable decrease in the intensity of the characteristic peaks of serpentinite and the appearance of new peaks characteristic of forsterite and diopside. The temperature of 660 °C in the derivatogram is in the region of the dehydroxylation of Mg_3_Si_2_O_5_(OH)_4_ and is the maximum point of this high-temperature process. The study of this process using NMR spectroscopy allowed us to conclude that the thermal processes with the formation of enstatite and forsterite occurred through the formation of dehydroxylate I (at 700 °C) and dehydroxylate II (at 800 °C) [20]. Later studies [16] indicated that forsterite, which was conventionally called low-temperature forsterite, can form at relatively low temperatures (600–700 °C), and high-temperature forsterite is formed at higher temperatures (800–830 °C).

**Figure 7:**
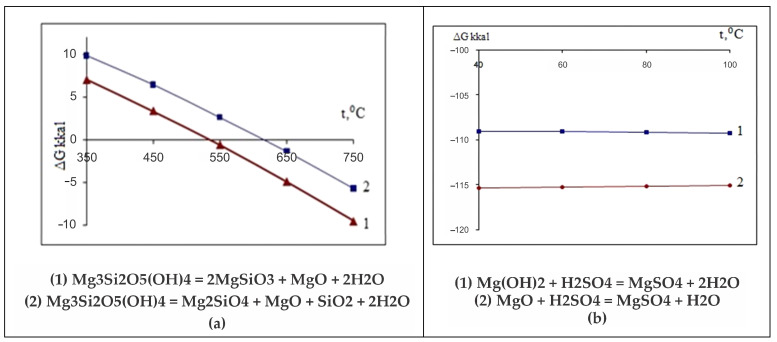
Thermodynamic scheme of thermal regulation of serpentinite with the formation of periclase (MgO) (**a**) and substituents MgO, Mg(OH)_2_ and H_2_SO_4_ (**b**).

Based on the analysis of diffractograms of samples No. 4 (660 °C), No.5 (725 °C) and No.6 (750 °C), it can be assumed that the beginning of the process of dehydroxylation of the brucite layer and the rupture of relatively unsaturated Si–O(Si) bonds in the siloxane (Si–O–Si) bridges of the hexagonal silicate Mg_3_Si_2_O_5_(OH)_4_ layer can be assumed to be at approximately 600 °C. The appearance of forsterite reflexes on the diffractogram of sample No. 4 (660 °C) indicates that regenerated orthosilicate and metasilicate anions, when they are in an amorphous crystalline state, can initiate the formation of forsterite microcrystals and the formation of amorphous enstatite, accompanied by the formation of periclase and tridymite, which are also found on the diffractograms of samples No. 4, No. 5 and No. 6. This assumption, at least, does not contradict the data obtained by us during the thermodynamic assessment of the Mg_3_Si_2_O_5_(OH)_4_ dehydroxylation process for the reactions shown in Figure 7a.

**Figure 8:**
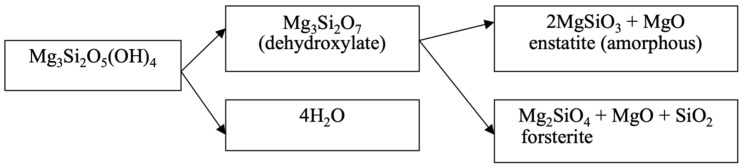
Diagram of periclase formation during thermal activation of serpentinite in the temperature range 600–750 °C.

The Gibbs energy dependence ∆G = f(T) shows that the probability of these reactions actually lies in the temperature range above 550 °C. It is obvious that, in reality, the reactions (Figure 7a) in the temperature range of 550–750 °C are carried out in sequence (Figure 8).

Since the processes of dehydroxylation and destruction of the serpentinite crystal lattice at these temperatures occur in the presence of impurity minerals containing iron, aluminum and others, they can also participate in the formation of new solid-phase compositions, which is confirmed by the diffractograms of samples Nos. 4, 5, 6 and 7. The intensity of the pyrope, almandine and magnetite reflexes increased with an increase in the firing temperature, and the intensity of the periclase reflexes at 725 °C and 750 °C were almost the same. According to [21,22], enstatite (amorphous) crystallizes at t > 807 °C.

Of the discovered mineral phases that exist separately in the thermolysis product of this serpentinite (up to 800 °C) (periclase, pyrope, almandine, forsterite, magnetite, diopside), all except periclase are insoluble or slightly soluble in sulfuric acid. Magnesium oxide is more soluble in water and has a stronger alkaline property than brucite and brucite-like components in Mg_3_Si_2_O_5_(OH)_4_, as indicated by the data of the thermodynamic evaluation of their reactions with sulfuric acid (Figure 7a).

**Figure 9:**
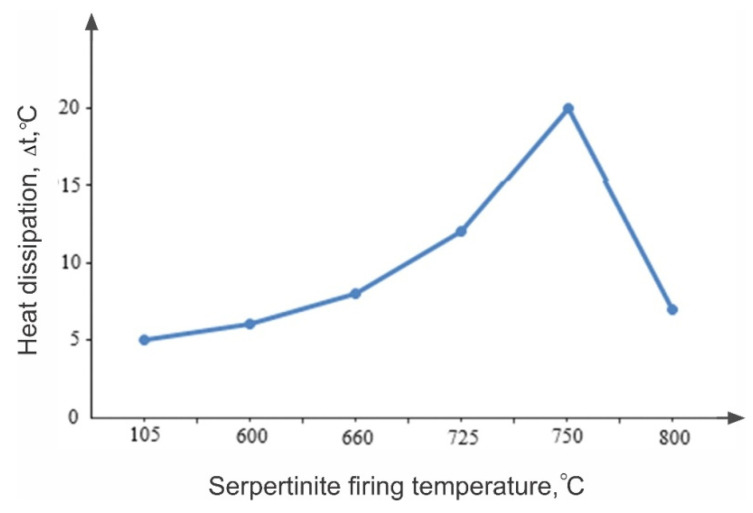
Dependence of heat release during dissolution of thermally activated serpentinite in 1.0 M H_2_SO_4_ on the heat-treatment temperature.

Observations of the comparative nature of the dissolution of thermally activated serpentinite (10.0 g each) (Figure 9) and chemical analysis of the quantitative transition of magnesium ions (Table 2) into a sulfate solution at their dissolution of 1.0 M H_2_SO_4_ (V_const_ = 100 mL) show that, when sample No. 1 (105 °C) was added to the acid solution, the suspension temperature rose from 24 °C to 27 °C. However, when adding No. 3 (600 °C), the temperature rose to 85 °C, for No. 4 (660 °C) to 88 °C and for No. 5 (725 °C) to 92 °C, and when adding No. 6 (750 °C), the interaction with the acid solution occurred violently with boiling, with the temperature rising to 98 °C. The degree of magnesium transition from serpentinite to solution increased from 40.0 % (No. 1, 105 °C) to 86.3% (No. 6, 750 °C).

The results of these observations and studies undoubtedly indicate that the firing temperature of serpentinite, during the ongoing physico-chemical changes and phase transformations, has a strong influence on the nature of the dissolution of serpentinite in acid solutions. Especially noteworthy is the fact of an increase in the alkaline properties of serpentinite during its firing in the temperature range of 600–750 °C, which has a significant effect on the kinetic characteristics of the dissolution of serpentinite and the extraction of magnesium ions into a sulfate solution.

## 3. Materials and Methods

Methodology for selecting the temperature of thermal activation (and calcination) of serpentinite.

**Figure 10:**
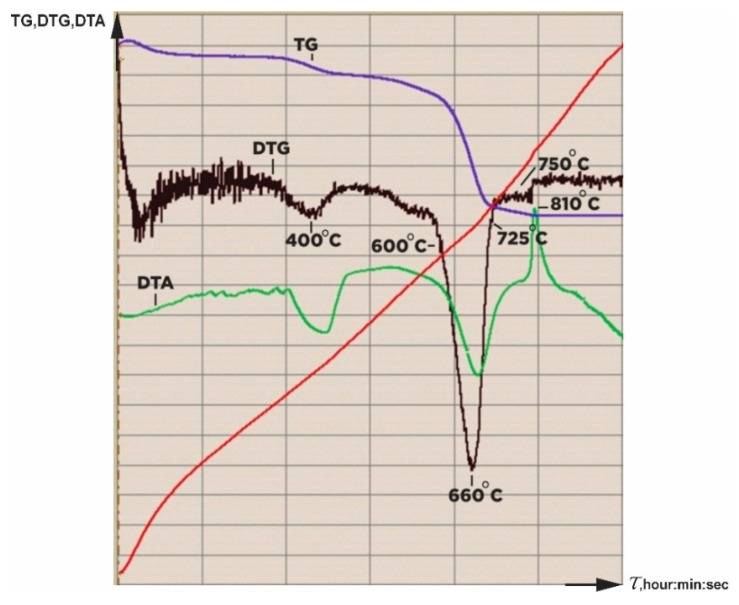
Derivatogram of the initial serpentinite.

At the beginning of this study, a derivatogram of the serpentinite under study was captured (Figure 10). According to the derivatogram, four areas were identified in which significant changes occurred during the calcination of serpentinite: I—temperature range with max 105 °C, removal of adsorbed moisture; II—temperature range 350–450 °C with max 450 °C, decomposition of brucite; III—temperature range 600–750 °C with max 660 °C, where the integrity of the serpentinite structure of the crystal lattice Mg_3_Si_2_O_5_(OH)_2_ is being destroyed; IV—temperature range of 750–800 °C for the formation of forsterite. As a result, seven serpentinite samples were selected for the study of thermal activation at the following temperatures: 105 °C (No. 1), 400 °C (No. 2), 600 °C (No. 3), 660 °C (No. 4), 725 °C (No.5), 750 °C (No. 6) and 800 °C (No. 7). To prepare the samples for study, the initial crushed serpentinite (d < 1.0 mm), weighing 1500 g, was dried at 105 °C in a drying cabinet to a stable weight. Then, seven samples of 100 g each were prepared from dried serpentinite and individually calcined for 1 h at the selected thermal activation temperatures.

Thermal curves (TG, DTA) were taken on the device “Q-DERIVATOGRAPH (Hungary) (System: F. Paulik, J. Paulik, L. Erdey).

X-ray images were taken on a D8Advance (“Bruker Optik GmbH”, Karlsruhe, Germany) device, Cu-Ka, tube voltage 40 kV, current 40 mA. The processing of the obtained diffractograms and the calculation of interplane distances were carried out using EVA software (DIFFRAC.EVA, V4). The decoding of samples and the search for phases were carried out using the Search/match program using the PDF-2 Powder Diffractometric Database (JCDD) and information provided in [23].

The chemical analysis was performed on a JSM-6490LV device, JEOL (Tokyo, Japan), complete with INCAEnergy 350 energy dispersive microanalyzer systems.

## 4. Conclusions

Heat treatment up to 600 °C did not affect the integrity of the layered structural composition of the serpentinite crystal lattice, which could lead to a significant change in its physico-chemical properties. The destruction of the structure of the serpentinite crystal lattice occurred in the temperature range of 600–800 °C. At the same time, the destruction of the structural composition of Mg_3_Si_2_O_5_(OH)_4_ was almost completed at a firing temperature of 750 °C. The occurrence of periclase and low-temperature forsterite in the composition of serpentinite fired at 660–750° is the main reason for the thermal activation of serpentinite, leading to an increase in its reactivity in relation to the effects of acids.

Therefore, the thermal activation of serpentinite (up to 750 °C) before its acid leaching may be one of the key positions in the development of new acidic methods for processing serpentinite and serpentinite waste accumulated during the processing of chrysotile asbestos ores at the locations of various deposits.

The results of this study show the prospects for continuing research in this area, following the use of serpentinite from the Kazakhstani deposit to produce industrially important magnesium compounds.

This study was conducted with the financial support of the Scientific Research Center of the Ministry of Education and Science of the Republic of Kazakhstan (BR21882242).

## Figures and Tables

**Table 1:** Conditions and weight loss during calcination of serpentine, τ = 1 h.

Sample No.	Calcination Temperature, °C	Process Area	Extreme Calcination Points	Serpentine Mass, g	Weight Loss, g
1	105	I—removal of adsorbed water	max	100	1.15
2 *	400	II—decomposition of brucite	max	100	2.58
3	600	III—decomposition of serpentinite	beginning	100	5.24
4	660		max	100	11.38
5	725		ending	100	13.34
6	750	IV—forsterite formations	beginning	100	13.77
7	800		max	100	13.92

* Diffractogram is not provided.

**Table 2:** The degree of transition of Mg into solution during dissolution of serpentinite in 1.0 M H_2_SO_4_, thermally activated at (105–800°), at t = 80 °C.

Sample No.	Processing Temperature, °C	Degree of Conversion of Magnesium into Sulfate Solution in % (1.0 M H_2_SO_4_)
1	105	40.0
2	400	41.4
3	600	46.6
4	660	72.9
5	725	82.3
6	750	86.3
7	800	81.16

## Data Availability

The data used to support the findings of this study are included within the article.

## References

[1] Yoo K., Kim B.-S., Kim M.-S., Lee J., Jeong J. (2009). Dissolution of Magnesium from Serpentine Mineral in Sulfuric Acid Solution. Mater. Trans..

[2] Sirota V., Selemenev V., Kovaleva M., Pavlenko I., Mamunin K., Dokalov V., Yapryntsev M. (2018). Preparation of Crystalline Mg(OH)_2_ Nanopowder from Serpentinite Mineral. Int. J. Min. Sci. Technol..

[3] Punenkov S.E., Kozlov Y.S. (2022). Chrysotile Asbestos and Resource Conservation in the Chrysotile Asbestos Industry. Min. J. Kazakhstan.

[4] U.S. Department of Health and Human Services (2011). 12th Report on Carcinogens.

[5] Dikanbayeva A.K., Auyeshov A.P., Satayev M.S., Pirminova I.V., Yeskibayeva C.Z., Arynov K.T. (2022). Influence of Structural and Molecular Features of Chrysotile on Interaction with in Acid-Chrysotile System. Rasayan J. Chem..

[6] Aueshov A.P., Satimbekova A.B., Arynov K.T., Dikanbaeva A.K., Bekaulova A.A. (2020). Environmental and Technological Aspects of Acid Treatment of Serpentinite Waste from Chrysotile Asbestos Mining and Processing. Int. J. Eng. Res. Technol. (IJERT) India.

[7] Aueshov A.P., Arynov K.T., Yeskibaeva C.Z., Aueshov A.A., Ibraeva A.M., Alzhanov K.B., Satimbekova A.B. (2018). Method for Processing Serpentinite. Patent for Utility Model.

[8] Taubert L. (2000). Hydrochloric Attack of Serpentinites: Mg^2+^ Leaching from Serpentinites. Magnes. Res..

[9] Gladikova L., Teterin V., Freidlina R. (2008). Production of Magnesium Oxide from Solutions Formed by Acid Processing of Serpentinite. Russ. J. Appl. Chem..

[10] Nagamori M., Boivin J.A. (2001). Technico-economic Simulation for the HCl-leaching of Hybrid Serpentine and Magnesite Feeds. Can. Metall..

[11] Auyeshov A., Arynov K., Yeskibayeva C., Dikanbayeva A., Auyeshov D., Raiymbekov Y. (2024). Transformation of Silicate Ions into Silica under the Influence of Acid on the Structure of Serpentinite. Molecules.

[12] Yeskibayeva C., Auyeshov A., Arynov K., Dikanbayeva A., Satimbekova A. (2024). Nature of serpentinite interactions with lowconcentrationsulfuric acid solutions. Green Process. Synth..

[13] Auyeshov A., Satimbekova A., Arynov K., Bekaulova A., Yeskibayeva S., Idrisheva Z. (2021). Environmentally friendly and resource-saving technology for disposal of dusty asbestos-containing wastes and production of magnesium salts. ARPN J. Eng. Appl. Sci..

[14] Fedoročková A., Plešingerová B., Sučik G., Raschman P., Doráková A. (2014). Characteristics of Amorphous Silica Prepared from Serpentinite Using Various Acidifying Agents. Int. J. Miner. Process..

[15] Zulumyan N.O., Oganesyan E.B., Oganesyan Z.G. (2002). On the Thermoacid Treatment of Serpentinites from the Northeastern Coast of Lake Sevan. Rep. Natl. Acad. Sci. Repub. Armen. Inorg. Chem..

[16] Papakhyan L.R., Terzyan A.M., Isaakyan A.R., Zulumyan N.O. (2014). Study of Lizardite Thermolysis from the Monti-Livornosi Deposit (Italy). Bull. State Eng. Univ. Armen. Ser. Chem. Environ. Technol..

[17] Fedoročková A., Hreus M., Raschman P., Sučik G. (2012). Dissolution of Magnesium from Calcined Serpentinite in Hydrochloric Acid. Miner. Eng..

[18] Zulumyan N.O., Isaakyan A.R., Oganesyan Z.G. (2007). A New Perspective Method of Serpentinite Processing. J. Appl. Chem..

[19] Maslennikova T.P. (2012). Study of the Chemical Interaction Processes of Hydrosilicate Nanotubes with Aqueous Solutions of Hydroxides and Salts of Alkali Metals (Na, K, Cs) and Aqueous-Alcohol Solutions (RCH_2_-OH). Ph.D. Thesis.

[20] Mackenzie K.J.D., Meinhold R.H. (1994). The Thermal Reactions of Talc Studied by 29 Si and 25 Mg MAS NMR. Thermochim. Acta..

[21] Rham dhani M.A., Hayes P.C., Jak E. (2012). Nickel Laterite. Part 1. Microstructure and Phase Characterizations During Reduction Roasting and Leaching. Miner. Process..

[22] Błońska E., Januszek K., Małek S., Wanic T. (2016). Effects of Serpentinite Fertilizer on the Chemical Properties and Enzyme Activity of Young Spruce Soils. Int. Agrophys..

[23] Mikheev V.I., Saldau E.P. (1965). X-ray Mineral Determinant.

